# AMPA and NMDA receptor antibody autoimmune encephalitis preceded by ocular myasthenia gravis: a case report

**DOI:** 10.1186/s12883-023-03129-2

**Published:** 2023-03-10

**Authors:** Jakob Schäfer, Peter Brøgger Christensen, Kimmo Jensen

**Affiliations:** 1 Department of Neurology, Aalborg University Hospital, Aalborg, Denmark; 2 Department of Clinical Medicine, Aalborg University, Aalborg, Denmark

**Keywords:** Autoimmune encephalitis, Myasthenia gravis, Cerebrospinal fluid, Treatment

## Abstract

**Background:**

α-Amino-3-hydroxy-5-methyl-4-isoxazole propionic acid (AMPA) and N-methyl-D-aspartate (NMDA) receptors mediate excitatory neurotransmission in the brain and may be targeted by autoantibodies, leading to autoimmune synaptic encephalitis (AE). AE can be associated with other autoimmune diseases. However, the cooccurrence of anti-AMPA and NMDA receptor AE together with myasthenia gravis (MG) is unusual.

**Case presentation:**

A 24-year-old previously healthy male presented with seronegative ocular MG, the diagnosis of which was supported by single-fiber electrophysiology findings. Three months later, he developed AE, initially being positive for AMPA receptor antibodies and subsequently for NMDA receptor antibodies. No underlying malignancy was found. In response to aggressive immunosuppressive treatment, he recovered (modified Rankin Scale (mRS) score change from 5 to 1). Despite some cognitive problems at the 1-year follow-up, which were not revealed using the mRS, he was able to return to his studies.

**Conclusions:**

AE may coexist with other autoimmune disorders. Patients with seronegative MG, including ocular MG, may develop autoimmune encephalitis with more than one cell-surface antibody.

## Background

Regarding autoimmune encephalitis (AE), most patients have only one specific antibody defining a syndrome, which in most cases can be identified by the clinical features, MRI findings, and cerebrospinal fluid changes [1]. For example, anti-AMPAR AE usually presents as a loss of short-term memory together with classic MRI findings consisting of hyperintensities restricted to the medial temporal lobes [2]. Rarely, a second immune response can be detected in cerebrospinal fluid (CSF) or in serum, and the clinical importance of such findings is uncertain. A recent study has shown that 4–7.5% of patients with anti-NMDAR encephalitis have concurrent glial or neuronal surface antibodies, which may influence the prognosis [3]. We report the case of a young male presenting with ocular myasthenia gravis (MG) who subsequently shortly thereafter developed severe anti-AMPA and anti-NMDA receptor encephalitis with clinical features of both conditions.

## Case presentation

A 24-year-old healthy male without a previous autoimmune disease history presented with diplopia and eye lid ptosis for 2 weeks. During the subsequent admission, he developed left-sided ptosis and impaired eye movements. The findings of the neurological examination were otherwise normal. The findings of MRI of the brain and orbits as well as a routine CSF analysis were normal. Jolly test results were positive. Test results for MG-associated AChR antibodies, calcium channels, Titin, and MuSK were all negative. However, single-fiber electromyography (EMG) showed jitter and decrement in the m. orbicularis oculi, suggesting seronegative ocular MG. Treatment with pyridostigmine and oral prednisolone improved the symptoms.

**Fig. 1:**
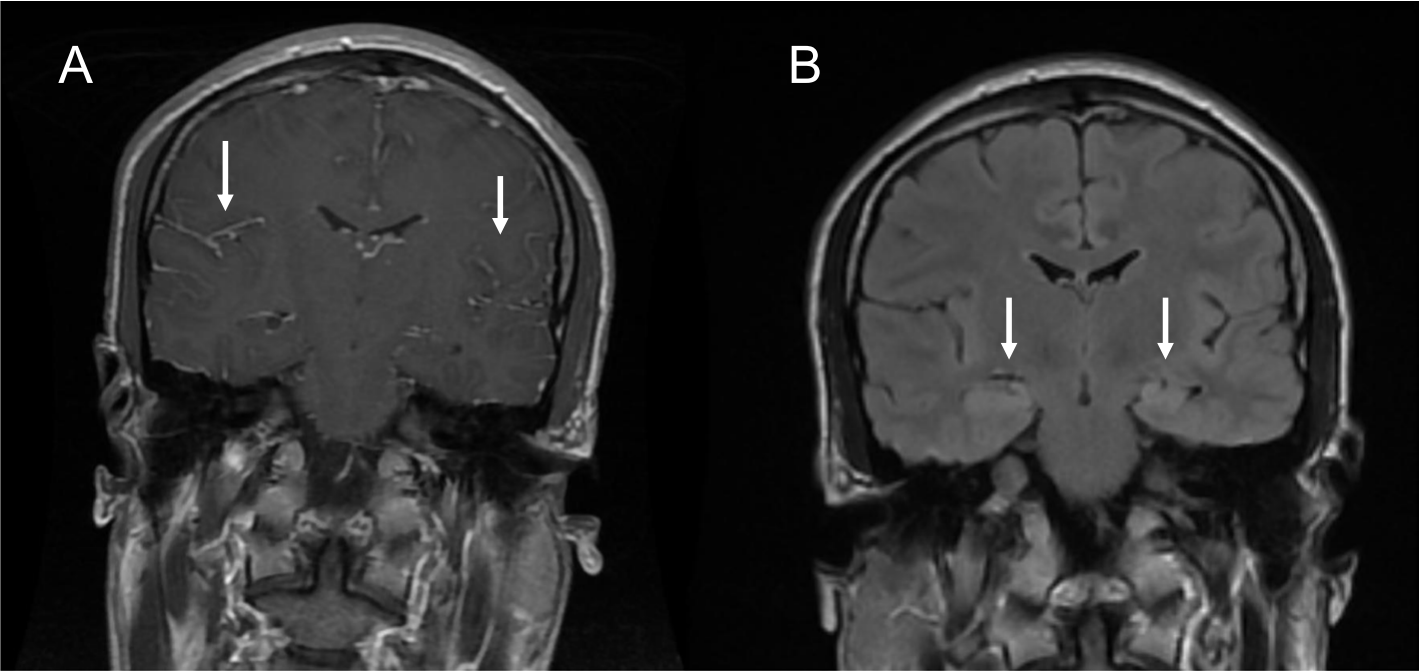
**A** Coronal brain MRI T1-weighted images with gadolinium contrast on April 13 (Day 0) demonstrate leptomeningeal enhancement (arrows). These findings were transient and were not demonstrated on an MRI scan on April 19 (Day 7). Usually, leptomeningeal enhancement is suggestive of infection, vasculitis or neurosarcoidosis; however, the results of a comprehensive screening for these conditions were normal. It was particularly important to exclude herpes CNS infection (HSV 1, HSV 2, HHV 6,7) because these infections can precede or coexist with AE [4]. **B** Coronal brain MRI T2-weighted FLAIR images on April 19 (Day 7) demonstrate bilateral hyperintensities in the medial temporal region (arrows), suggesting limbic encephalitis

Three months later, he complained about a loss of short-term memory, behavioral changes, fatigue, depressed mood, and unsteady gait. At readmission (Day 0), he was awake but disoriented with a loss of short-term memory. He had bilateral ptosis, his gait was unstable, and he had dystonic posture of the feet. MRI of the brain showed leptomeningeal contrast enhancement, compatible with inflammation or vasculitis (Fig. 1A). CSF examination showed pleocytosis with 63 mononuclear cells, normal protein, signs of intrathecal IgG synthesis (IgG index 0.92; range 0.80–0.91), and the detection of oligoclonal bands (Table 1). Electroencephalography (EEG) findings were normal. We initiated acute treatment with acyclovir 10 mg/kg i.v. 3 times daily and ceftriaxone 2 g i.v. twice daily, as well as high-dose i.v. methylprednisolone 1 g daily. A repeated CSF examination on Day 4 showed a strong positive reaction for AMPAR in serum and CSF, while the reactions for other autoimmune encephalitis antibodies, including NMDAR antibodies and paraneoplastic antibodies, were negative. Analyses of AE antibodies (CASPR2, GABA-B, GAD65, AMPA, LGI1, NMDA, DPXX, GABA-A, IgLON) and paraneoplastic antibodies (Amphiphysin, CDR2, DRP-5, Hu, PNMA2, NOVA1, GAD65, Recoverin, SOX-1, DNER, Zic 4, Titin) were performed by an accredited laboratory (Dept. of Clinical Immunology, Odense University Hospital, Denmark) using indirect immunofluorescence tests (Euroimmun, Lübeck, Germany). Positive results were confirmed in a tissue-based assay. Results for neuroinfection screening at that time (i.e., PCR in CSF: Herpes simplex virus 1 (HSV-1), Herpes simplex virus 2 (HSV-2), Varicella zoster virus (VZV), Human herpes virus 6 and 7 (HHV 6, HHV 7), Cytomegalovirus (CMV), Enterovirus, *Escherichia coli*, *Haemophilus influenzae*, *Listeria monocytogenes*, *Neisseria meningitis*, *Streptococcus agalactiae*, *Streptococcus pneumoniae*, *Cryptococcus neoformans*; CSF VDRL (syphilis); Borrelia-specific CSF/serum antibody index; and tick-borne encephalitis virus (TBE), HIV, *Mycobacterium tuberculosis* and fungal testing) were normal. In addition, vasculitis laboratory test results (CRP, ESR, ANA, ANCA, immunoglobins, complement, hepatitis serology, ACE and interleukin 2 receptor antibodies) were normal. Because CNS infection screening results were negative, acyclovir and ceftriaxone treatment was stopped on Day 4.

**Table 1:** Cerebrospinal fluid findings over time in the patient

CSF composition	Jan 20 2018	Apr 12 2018	Apr 16 2018	May 25 2018	June 26 2018	Feb 8 2019	May 9 2019	Mar 27 2020
**Leucocytes** (poly/mononuclear)	3 (1/2)	**66** (3/63)	**64** (2/62)	5 (1/4)	1 (0/1)	2 (0/2)	4 (0/4)	2 (0/2)
**Protein** (0.15–0.45 g/L)	0.43	0.43	0.26	0.42	0.52	0.35	0.37	0.28
**IgG index** (0.80–0.91)	0.87	**0.92**	**1.01**	0.87	0.87	0.85	0.87	0.87
**Oligoclonal bands**	N.D.	**Detected**	**Detected**	N.D.	N.D.	N.D.	N.D.	N.D.

Abnormal values are shown in bold. *CSF* Cerebrospinal fluid, *N.D.* Not detected

**Fig. 2:**
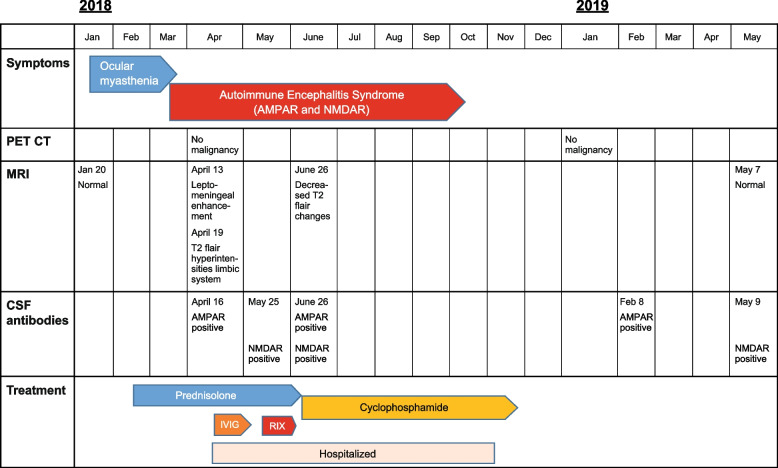
Overview of the clinical course and treatment of autoimmune encephalitis in a 24-year-old male. While initially testing positive for AMPA receptor antibodies and subsequently for NMDA receptor antibodies, the patient tested positive for both AMPA and NMDA receptor antibodies approximately 3 months after his clinical presentation of encephalitis symptoms. CSF: cerebrospinal fluid, IVIG: intravenous immunoglobulin treatment, RTX: rituximab

A repeated MRI of the brain on Day 7 after readmission showed bilateral signal increases in T2 FLAIR sequences in the limbic system and insula (Fig. 1B). As limbic encephalitis was suspected, treatment with high-dose i.v. steroid of 1 g methylprednisolone daily for 3 days was resumed, followed by treatment with intravenous immunoglobin 0.4 g/kg/daily (IVIg) (two 5-day treatments) and rituximab (4 courses of 375 mg/m^2^) once a week. Due to the poor response, an additional fifth dose was given. Further clinical deterioration was noted, with psychomotor agitation and disorientation as well as bulbar symptoms and orofacial dyskinesia. This rapid progression of symptoms led to admission to the intensive care unit, and at this point (Day 43), the patient tested positive for NMDAR antibodies in CSF and serum. He was then sedated and connected to a ventilator for 3 days. After 4 days, his condition became stable, and he was extubated. EEG at that time showed diffusely reduced amplitudes but no sign of status epilepticus and no focal abnormalities. After an initial favorable response to treatment, the patient’s psychiatric symptoms increased, and he was admitted to a psychiatric department in a psychotic state. Due to generalized tonic clonic seizures, anticonvulsive medication with lacosamide and valproic acid was initiated. After this deterioration, third-line immunosuppressive treatment was initiated and continued with pulses of cyclophosphamide 750 mg/m^2^ i.v. once a month for a duration of 6 months (Fig. 2).

An extensive diagnostic program could not determine any proof of infection, vasculitis or malignancy. A CT scan of the thorax and abdomen showed some remaining thymus, but there were no signs of malignancy, even though microthymoma could not be ruled out. A PET-CT scan showed unspecific activity in the axillary lymph nodes. Due to the myasthenic component of the illness, endoscopic thymectomy was performed. However, no pathology was noted in the patient’s thymus or lymph nodes.

The patient gradually recovered during several months of hospitalization, after which he was transferred to a neurorehabilitation unit. At the 1-year follow-up, we observed an almost complete recovery (mRS score change from 5 to 1), but a comprehensive neuropsychological assessment showed mild to moderate reduced verbal and nonverbal episodic memory. His executive abilities were slightly impaired (abstraction, reasoning, flexibility). His everyday life had to be very structured, and he needed the support of his girlfriend and his teachers at university. He had amnesia regarding the period of hospitalization. A repeated PET-CT scan showed no signs of malignancy, and EEG findings were normal. His cognitive symptoms were in remission, and he was able to return to his engineering studies.

## Discussion and conclusions

The present case is typical for AE, and most of the aggressive clinical picture is compatible with anti-NMDAR AE [5, 6]. The additional MRI findings, such as bilateral mesial temporal hyperintensities, suggest the occurrence of more than one antibody, e.g., in our case, AMPAR. MRI at Day 0 showed leptomeningeal enhancement, which is unusual in AE, although early transient enhancement, as in our case, can occur [7]. Normally, leptomeningeal enhancement is suggestive of infection, vasculitis or neurosarcoidosis, but the results of comprehensive screening for these conditions were normal. It is particularly important to exclude HSV and HHV infection since these infections can precede or coexist with AE [4].

A recent study showed that 4–7.5% of patients with anti-NMDAR encephalitis have concurrent glial or neuronal surface antibodies, which may influence the prognosis [3]. Among the 848 patients in this cohort, 6 had NMDAR and AMPAR antibodies, and five of these had cancer, in contrast to our case. The patient without cancer had a clinical course very similar to that of our patient. In a case series of 22 AMPA antibody-positive patients, four had thymoma, but MG was not reported, while seven had additional neuronal antibodies, of which two were double-positive for AMPAR and NMDAR antibodies [8]. In this report, there were no details on the clinical course. In two other single cases, an association between AMPAR AE and thymomatous MG was reported [9, 10], but in our patient, thymoma was not detected.

Although AMPAR antibodies preceded the presence of NMDA antibodies (Fig. 2), our patient had clinical features of both conditions: a loss of short-term memory and MRI findings suggesting a diagnosis of anti-AMPAR encephalitis, and severe psychosis, seizures, and movement abnormalities, including oral dyskinesia, which fulfilled the diagnostic criteria for anti-NMDAR encephalitis.

Most patients with anti-AMPAR encephalitis show limbic encephalitis, accompanied by the presence of neoplasms in more than 50% of cases (SCLC, thymoma, breast cancer) [11]. A loss of short-term memory and bilateral hyperintensities restricted to the medial temporal lobes are hallmarks of this disorder. Some patients present with fulminant encephalitis, while others present with psychosis or seizures. Most patients experience only a partial recovery. On the other hand, anti-NMDAR encephalitis, which is much more common, is characterized by a complex neuropsychiatric syndrome, seizures, abnormal movements, oral dyskinesia and a decreased level of consciousness [12]. Findings of MRI of the brain are either normal or show nonspecific abnormalities, including transient leptomeningeal enhancement [7]. The frequency of associated cancer is low [13, 14], and most patients fully recover. However, it is important to be aware of any cognitive problems in the follow-up of the patients [15].

It is likely that the presence of both AMPA and NMDAR antibodies was responsible for the severely prolonged diphasic course in our patient. We therefore suggest that the presence of more than one antibody should be considered in patients with atypical autoimmune encephalitis. The reason why some patients have two or more autoimmune diseases is not known, but an association with HLA class II alleles may play a role [5, 16]. The outcome in our patient was better than expected (mRS score change from 5 to 1), which emphasizes the importance of aggressive immunosuppressive treatment. In accordance with the findings of a previous study [15], our patient had cognitive problems at the follow-up that were not revealed using the mRS. Finally, in a recent study of 517 patients, it was concluded that the occurrence of autoimmune comorbidities such as MG did not alter the clinical course of AE [17].

In conclusion, patients with seronegative MG, including ocular MG, may develop autoimmune encephalitis with more than one cell-surface antibody. One may expect these findings only in MG-seropositive patients.

## Data Availability

The datasets used and/or analyzed during the current study are available from the corresponding author on reasonable request.

## Abbreviations

ACE: Angiotensin converting enzyme
AChR: Acetylcholine receptor
AE: Autoimmune encephalitis
AMPA: α-Amino-3-hydroxy-5-methyl-4-isoxazole propionic acid
ANA: Antinuclear antibodies
ANCA: Antineutrophil cytoplasmic antibodies
CASPR2: Contactin associated protein 2
CDR2: Cerebellar degeneration-related protein 2
CMV: Cytomegalovirus
CRP: C-reactive protein
CSF: Cerebrospinal fluid
DNER: Delta and Notch-like epidermal growth factor-related receptor
DPXX: Dipeptidyl-peptidase-like protein-6
DRP-5: Collapsin response mediated protein 5
EEG: Electroencephalography
EMG: Electromyography
ESR: Erythrocyte sedimentation rate
FDG-PET: Fluorodeoxyglucose (FDG) positron emission tomography (PET)
GABA-A: Gamma-aminobutyric acid A
GABA-B: Gamma-aminobutyric acid B
GAD65: Glutamic acid decarboxylase
HHV 6: Human herpes virus 6
HHV 7: Human herpes virus 7
HIV: Human immunodeficiency virus
HLA: Human leukocyte antigen
HSV-1: Herpes simplex virus 1
HSV-2: Herpes simplex virus 2
IgLON: Neuronal cell adhesion molecule
IVIg: Intravenous immunoglobin
LGI1: Leucine-rich glioma-inactivated protein 1
MG: Myasthenia gravis
MRI: Magnetic resonance imaging
mRS: Modified Rankin Scale
NMDA: N-Methyl-D-aspartate
NOVA1: RNA-binding protein. A member of the Nova family of paraneoplastic disease antigens
PCR: Polymerase chain reaction
PNMA2: Paraneoplastic Ma family 2
SCLC: Small cell lung carcinoma
SOX-1: Sry-like high mobility group box 1
VDRL: Venereal Disease Research Laboratory
Zic 4: Zinc-finger protein 4

## References

[1] Blinder T, Lewerenz J (2019). Cerebrospinal fluid findings in patients with autoimmune encephalitis - a systematic analysis. Front Neurol.

[2] Lai M, Hughes EG, Peng X, Zhou L, Gleichman AJ, Shu H (2009). AMPA receptor antibodies in limbic encephalitis alter synaptic receptor location. Ann Neurol.

[3] Martinez-Hernandez E, Guasp M, García-Serra A, Maudes E, Ariño H, Sepulveda M (2020). Clinical significance of anti-NMDAR concurrent with glial or neuronal surface antibodies. Neurology..

[4] Linnoila JJ, Binnicker MJ, Majed M, McKeon A (2016). CSF herpes virus and autoantibody profiles in the evaluation of encephalitis. Neurol Neuroimmunol Neuroinflamm.

[5] Dalmau J, Graus F (2018). Antibody-mediated encephalitis. N Engl J Med.

[6] Titulaer MJ, McCrachen L, Gabilondo I, Armanqué T, Glaser C, Iizuka T (2013). Treatment and prognostic factors for long-term outcome in patients with anti-*N*-Methy-D-aspartate (NMDA) receptor encephalitis: a cohort study. Lancet Neurol.

[7] Dalmau J, Gleichmann AJ, Hughes EG, Rossi JE, Peng X, Lai M (2008). Anti-NMDA-receptor encephalitis. Case series and analysis of the effecs of antibodies. Lancet Neurol.

[8] Höftberger R, van Sonderen A, Leypoldt F, Houghton D, Geschwind M, Gelfand J (2015). Encephalitis and AMPA receptor antibodies: novel findings in a case series of 22 patients. Neurology..

[9] Li X, Mao Y-T, Wu J-J, Li L-X, Chen X-J. Anti-AMPA receptor encephalitis associated with thymomatous myasthenia gravis. J Neuroimmunol. 2015;281:35–7. 10.1016/j.jneuroim.2015.02.011. 10.1016/j.jneuroim.2015.02.011 25867465

[10] Luo Q, Wu X, Huang W. Anti-α-amino-3-hydroxy-5-methyl-4-isoxazolepropionic acid receptor GluR2 encephalitis in a myasthenia gravis patient with complete thymectomy: a case report. BMC Neurol. 2019;13;19(1):126. 10.1186/s12883-019-1358-7. 10.1186/s12883-019-1358-7 PMC6563362 31195997

[11] Joubert B, Kerschen P, Zekeridou A, Desestret V, Rogemond V, Chaffois M-O, et al. Clinical spectrum of encephalitis associated with antibodies against the α-Amino-3-hydroxy-5-methyl-4-isoxazolepropionic acid receptor. JAMA Neurol. 2015:72(10):1163–9. 10.1001/jamaneurol.2015.1715. 10.1001/jamaneurol.2015.1715 26280228

[12] Dalmau J, Armangué T, Planagumà J, Radosevic M, Mannara F, Leypoldt F, et al. An update on anti-NMDA receptor encephalitis for neurologists and psychiatrists: mechanisms and models. Lancet Neurol. 2019:18(11):1045–57. 10.1016/S1474-4422(19)30244-3. 10.1016/S1474-4422(19)30244-3 31326280

[13] Nissen MS, Ørvik MS, Nilsson AC, Ryding M, Lydolph M, Blaabjerg M. NMDA-receptor encephalitis in Denmark from 2009 To 2019: a national cohort study. J Neurol. 2021. 10.1007/s00415-021-10738-9. 10.1007/s00415-021-10738-9 34351472

[14] Bost C, Chanson E, Picard G, Meyronet D, Mayeur M-E, Ducray F, et al. Malignant tumors in autoimmune encephalitis with anti-NMDA receptor antibodies. J Neurol. 2018;265(10):2190–200. 10.1007/s00415-018-8970-0. 10.1007/s00415-018-8970-0 30003358

[15] Finke C, Kopp UA, Prüss H, Dalmau J, Wandinger K-P, Ploner CJ (2012). Cognitive deficits following anti-NMDA receptor encephalitis. J Neurol Neurosurg Psychiatry.

[16] Muniz-Castrillo S, Vogrig A, Honnorat J. Associations between Hla and autoimmune neurological diseases with autoantibodies. Autoimmun Highlights. 2020. 10.1186/s13317-019-0124-6. 10.1186/s13317-019-0124-6 PMC7065322 32127039

[17] Zhao J, Wang C, Xu X, Zhang Y, Ren H, Ren Z (2019). Coexistence of autoimmune encephalitis and other systemic autoimmune diseases. Front Neurol.

